# Comparison between Different Activation Overvoltage Descriptions for Semiempirical Proton-Exchange Membrane Fuel Cell Models

**DOI:** 10.1021/acsomega.4c11603

**Published:** 2025-04-10

**Authors:** Leonardo Fortuna Carneiro, Nicolas Tadeu Domingues Fernandes, Esly Ferreira da Costa Junior, Tulio Matencio

**Affiliations:** † Postgraduate Program in Chemical Engineering, PPGEQ-UFMG, Federal University of Minas Gerais, Presidente Antônio Carlos Avenue, 6627, Pampulha, Belo Horizonte, Minas Gerais 31270-901, Brazil; ‡ Postgraduate Program in Mechanical Engineering, PPGMEC-UFMG, Federal University of Minas Gerais, Presidente Antônio Carlos Avenue, 6627, Pampulha, Belo Horizonte, Minas Gerais 31270-901, Brazil; § Department of Chemistry, DEQ/ICEX-UFMG, Federal University of Minas Gerais, Presidente Antônio Carlos Avenue, 6627, Pampulha, Belo Horizonte, Minas Gerais 31270-901, Brazil

## Abstract

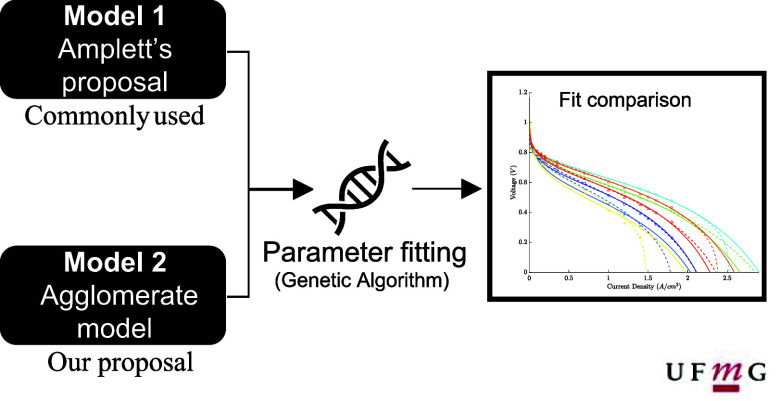

Using parameter estimation along with semiempirical fuel cell models is a promising strategy to obtain simple yet accurate models for proton-exchange membrane fuel cells. This work compares a model using a common semiempirical description of the activation overvoltage with one based on an agglomerate model. The parameters are estimated by nonlinear regression and the resulting optimization problem is solved using a parallel implementation of the genetic algorithm. The results indicate that the model incorporating the agglomerate description offers parameters with more straightforward physical interpretations and achieves superior fits to the experimental polarization data, both for individual curve fitting and when multiple curves are fitted simultaneously. Moreover, an analysis of the overvoltages shows that it is more robust in modeling reactant depletion effects at high current densities, attesting to its ability to represent relevant phenomena reasonably. Thus, further usage of parameter estimation along with agglomerate models is recommended as a useful strategy to describe fuel cells with limited data availability.

## Introduction

Proton-exchange membrane fuel cells (PEMFC) are currently regarded as promising energy-converting devices for a cleaner energy matrix.^1,2^ They have many remarkable qualities that justify this position, such as high efficiency, quiet operation, and no emissions in their energy conversion process.^3^ Although this technology has reached commercialization, many improvements are still required to reach their widespread usage.^4,5^ The high cost of the catalyst and insufficient multiphase transport process can be cited as examples of current limitations.^2,3^ Thus, a better understanding of these systems is necessary to achieve the established goals for the price, lifetime, and efficiency of these devices.

Besides the experimental analysis of PEMFCs, modeling is an important tool for studying fuel cells, being capable of providing valuable insights into mechanisms and transport phenomena that could be either too costly or hard to measure experimentally.^4,6^ Owning to this importance, many models for fuel cells have been developed, with different modeling approaches and levels of complexity. Usually, those capable of providing more details about the cell are complex 3D models. An example is the one developed by Ahmadi, Rezazadeh and Mirzaee,^7^ which was used to investigate the effect of some parameters on the cell’s behavior. Their results indicate the importance of the analyzed parameters in the cell’s efficiency and their interdependence, which are important information for the development of better systems. However, this complexity comes at the cost of high computational times, which can be prohibitive for some applications.^8^

Another major limitation of complex models is their dependence on many parameters that are normally not given by the manufacturer and are hard to measure. Usually, only basic information about the materials and a polarization curve at a specific condition are available for commercial fuel cells. This hinders their usage for dimensioning and optimizing systems for large-scale applications, especially when the cells are not available for tests. Thus, although complex models better represent the phenomena in a fuel cell, a more pragmatic approach is necessary for some engineering applications.

This necessity, along with the increasing availability of experimental data, led to a significant growth in the interest in data-driven PEMFC models, that is, those that do not use an explicit physical description of the fuel cell to evaluate their results.^4^ Although the main interest of this type of model is usually describing polarization curves, they are also promising for aiding in other descriptions. For example, Tian et al.^9^ presented a model capable of using generative inference output to effectively predict the long-term degradation of a PEMFC. Even though they complement well the shortcomings of phenomenological models, data-driven approaches also present many limitations. Besides not providing a physical interpretation behind their results, the high computational demands and lack of data covering crucial parameters for training them currently still hinder their applicability.^10^ So, although useful, they are still limited for many applications.

An alternative to contouring the challenges of both data-driven and complex phenomenological descriptions is the usage of semiempirical models along with parameter estimation. This type of model is still physics-based, so they may be useful to reach conclusions that are representative of most PEMFCs, but they have a considerably lower computational cost when compared to a phenomenological description of the transport phenomena. For example, Lu et al.^11^ employed this type of model in the analysis of the impact of high temperatures and pressures on the performance of a fuel cell stack, obtaining insightful results.

Between the semiempirical descriptions available, the model developed by Mann et al.^12^ is among the most commonly used. It describes the activation overvoltage using four semiempirical parameters proposed by Amphlett,^13^ assumed to be constant for a given cell, and the ohmic overvoltage is described as a function of current, temperature, and a parameter representing the membrane’s humidification. Many works estimate parameters for this model using different optimization techniques, such as particle swarm optimization,^14−17^ genetic algorithm,^18−20^ and many nature-inspired algorithms.^21−23^ Recent reviews about these techniques are provided by Priya^24^ and Mitra.^25^

Despite being used by many works, the application of Mann’s model to describe fuel cells has challenges related to the physical interpretation of the results and its descriptive capability of polarization data. First, the usage of parameters that lump many properties in the activation description makes their comprehension challenging, which may lead to meaningless values being used to describe a cell. This, for example, complicates the choice of boundaries when estimating them. Moreover, the accentuated increase in overvoltage at high current densities related to reactant depletion is not well described by Mann’s model. This has led most studies based on this approach to propose explicit modeling of the overvoltage related to transport effect (*η*_*conc*_) by eq 1, where *b* is an empirical parameter and *j*_*lim*_ is the limiting current density.
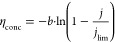
1

However, when a model already considers the reactants’ concentration at the reaction site – which is the case for many implementations of Mann’s model – the concentration effect is already considered.^26^ So, including an additional parameter to compensate for the insufficiency of the used description in capturing the full extent of the system’s losses may be detrimental to the physical meaning behind the fitted parameters.

Furthermore, the limiting current density is another point of incertitude because, although it should vary with operating conditions, it is normally assumed as a constant value by PEMFC models.^26^ This may limit the ability to predict results outside the specific conditions in which the parameters were fitted, as they represent mathematical values tailored to describe a specific data set rather than physically meaningful properties of the studied cell. Therefore, an approach with physically meaningful parameters that are capable of describing the whole range of polarization results is desirable.

A promising alternative is the usage of agglomerate models to describe the catalyst layer instead of thin-film or macro-homogeneous descriptions. This approach more closely describes the structure of the catalyst layer and, as a consequence, predicts experimental results better.^27^ It can effectively describe all regions of the polarization curve, even the sharp voltage drop at high current densities related to oxygen depletion.^28^ Thus, better results and more meaningful parameters may be obtained with its usage in semiempirical models.

Currently, some works have used parameter estimation along with an agglomerate model to describe polarization data. Secanell et al.^29^ used this technique to obtain the optimal composition for a cell’s cathode at different operating conditions. They used the catalyst loading, porosity of the layers, and the mass percentage of platinum catalyst on the support carbon black as variables for the proposed optimization problem. Another work using optimization strategies to characterize agglomerate model parameters is the one by Dobson et al.^30^ In it, the authors use polarization curves obtained at different conditions as a basis for fitting the agglomerate radius, agglomerate porosity, and the product between the catalyst’s active area and exchange current density. The model with optimized parameters was capable of accurately describing the data under multiple conditions when they were simultaneously considered in the optimization method, reinforcing the applicability of the proposed strategy to obtain good descriptions of the fuel cell’s properties and behaviors.

Nevertheless, a direct comparison between semiempirical models with different descriptions for the activation overvoltage has not been found in the literature. So, this work aims to evaluate if a semiempirical model with meaningful activation parameters based on an agglomerate model can provide better fits than the usual description based on Mann’s article, which is commonly used. This should provide valuable insights for future works using semiempirical models about which simplification level is satisfactory for the studied application. To do this, two models are developed and compared with experimental data under multiple conditions. A careful evaluation of the influence of each activation description is conducted, paying attention to their effect on the fit’s quality and in the optimal parameters. Both the assessment of these impacts and the proposal of a methodology for describing a semiempirical model with physics-based parameters that have clear meanings are innovations of this work that are expected to be useful for other researchers. Moreover, determining some limitations related to neglecting the agglomerate structure should also be a significant contribution of this work.

## Methodology

To quantify the differences between modeling approaches, it is first necessary to define the models’ objective. Here, given their practical importance, they are evaluated based on their ability to describe polarization data. More specifically, the polarization data of a single cell is considered, but the models could be adapted for a stack using the assumption that all cells in the stack operate at the same condition. Thus, in the following sections, the models and the methodology used to compare them are presented.

### Model Development

Two semiempirical models are developed for the proposed comparison. The first is an implementation based on the model by Mann et al.^12^ which uses Amphlett’s^13^ proposal to describe the activation overvoltage. The second follows this previous model’s structure but with an agglomerate model to describe the cathodic activation overvoltage. The basic assumptions valid for both are as follows:

The system is isobaric and isothermal.

All gases are ideal.

The catalyst layer thickness is much smaller than the gas diffusion layer one, so the concentrations inside it can be assumed to be homogeneous and equal to those at the catalyst layer/gas diffusion layer interface.

The system is monophasic, that is, water only exists as vapor.

The activation overvoltage in the anode can be neglected, as it is much smaller than the cathodic one.

Moreover, both assume that the cell’s current density is homogeneous, so its voltage can be obtained by subtracting the activation and ohmic overvoltages – *η*_*act*_ and *η*_*ohm*_ respectively – from the thermodynamic value, *E*_*thermo*_. This is represented in eq 2.
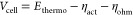
2

Among those assumptions, the two most critical are considering the system as isothermal and monophasic. The first may interfere with the predictions because most properties are temperature dependent, so it is still not clear to what extent the temperature variations that exist in a fuel cell impact the overall results. As for the other, flooding effects can be a significant part of the cause for overvoltage at certain conditions, especially at high current densities. So, the model is not expected to provide good descriptions when a significant amount of liquid water is present in the fuel cell.

The thermodynamic voltage is calculated with eq 3, Nernst’s equation.^12^ In it Δ*S*_*rxn*_ is the reaction’s entropy per mol of hydrogen reacted, equal to −163.076 J mol^–1^ K^–1^,^31^ and  and  are the partial pressures of hydrogen and oxygen at the catalyst layers. They are evaluated respectively by eq 4 and eq 5, obtained using Fick’s law.
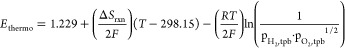
3
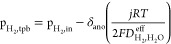
4
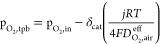
5

The effective diffusivity is evaluated as the product between the diffusion coefficient (*D*_*ij*_) and a microstructure factor *M*. eq 6, obtained in Chapman-Enskog kinetic theory of gases,^32^ is used to evaluate the diffusion coefficient, and its parameters are presented in Table 1. As for the microstructure factor, it is described here using eq 7, based on the straight-capillary-tube model, along with a correction to account for the liquid water saturation (*s*).^33^ This last parameter is assumed to be a constant value equal to the immobile saturation for the used gas diffusion layer because the model does not directly take into account the liquid phase.
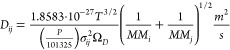
6
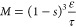
7

**Table 1:** Parameters Used in the Evaluation of the Diffusion Coefficient^a^

Gas	MM(kg kmol^–1^)	σ(m)	*ε*/*k* (K)
H_2_	2.016	2.92 × 10^–10^	38.0
H_2_O	18.015	2.73 × 10^–10^	355.7
O_2_	31.999	3.43 × 10^–10^	113.0
Air	28.964	3.62 × 10^–10^	97.0

a Source: refs. (32,34)

Moreover, the ohmic overvoltage is obtained using eq 8. In it,  is the area-specific resistance related to proton transport in the ionomer and *ASR*_*other*_ encompasses other ohmic resistances present in the system. The latter is a fitting parameter, and the former is evaluated by multiplying the ionomer’s resistivity – , given as a function of a fitting parameter representing the membrane humidification (λ) – by the thickness of the layers with the ionomer. This is shown in eqs 9 and 10, where it is highlighted that the thickness of the catalyst layers is also considered because the protons need to be transported in the ionomer present in them. Although commonly neglected, with the modern membranes having thicknesses similar to those of catalyst layers, not considering their effect may lead to a significant underestimation of the ohmic overvoltage. Note that the thickness of the catalyst layer was previously neglected when evaluating the concentration because there it is compared with the GDL, which is much thicker than the CL. Here, however, it is compared to the membrane, with similar thickness. Therefore, neglecting the CL thickness when evaluating the activation overvoltage is likely reasonable, but it may not be when evaluating the ohmic one.
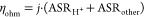
8
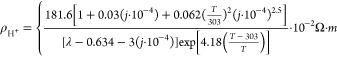
9
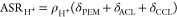
10

Finally, the activation overvoltage for Mann’s model is calculated using eq 11, where each ξ is a fitting parameter and  is the concentration at the triple-phase boundary, given by eq 12.^13,23^
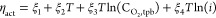
11
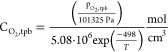
12

The agglomerate model, on the other hand, has a more complex description of this overvoltage. In contrast to the other approach, it demands knowledge of the catalyst loading and the ionomer content in the catalyst layer. The first information is commonly given by the manufacturers, but the second usually needs to be assumed if a commercial cell is used. Furthermore, the fitting parameters are chosen as values with a direct physical meaning, which considerably eases the choice of boundaries for them.

First, considering that the agglomerate’s pores are filled by ionomer, the volumetric fraction occupied by the solid phase (Pt/C) and the catalyst layer’s porosity can be obtained from eqs 13 and 14, respectively.^35^ In them, *f*_*Pt*_ is the platinum ratio in the Pt/C – used as a fitting parameter – , and *φ*_*i*,*CL*_ is the ionomer fraction at the catalyst layer.
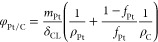
13
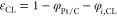
14

This parameter is also used to evaluate the catalyst-specific area () with an empirical formula proposed by Khajeh-Hosseini-Dalasm et al.,^36^ shown in eq 15. Here, *a*_*eff*_ represents the fraction of the initial area available after incorporation, *m*_*Pt*_ is the platinum loading, and *δ*_*CL*_ is the thickness of the catalyst layer.
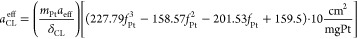
15

Using these values, it is possible to calculate the agglomerate density (eq 16), the thickness of the films covering the agglomerate (eqs 17 and 18), and the ionomer’s and water’s effective surface area (eqs 19 and 20). The agglomerate radius (*r*_*agg*_) and the volumetric fraction of ionomer in the agglomerate (*φ*_*i*,*agg*_) are used as fitting parameters. As for the liquid water saturation (*s*), the same value employed in eq 7 is utilized.
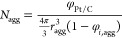
16
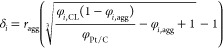
17
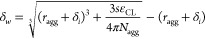
18
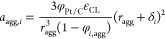
19
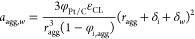
20

Then, the reaction constant can be calculated with eq 21, in which the Thiele modulus and the effectiveness factor for the spherical agglomerates are given by eqs 22 and 23.^35^ This constant depends on the transfer coefficient (*α*_*cat*_), used as the final fitting parameter, and the overvoltage itself. Moreover, the oxygen’s reference concentration () is equal to 2.6193 mol m^–3^, evaluated with Henry’s law at the same conditions of the reference exchange current density ().
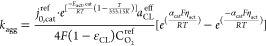
21
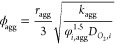
22
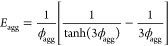
23

To determine the overvoltage, eq 24 is solved numerically for the given current density.^35,37^ The oxygen’s Henry constant and diffusivity in the ionomer are given by eqs 25 and 26,^38^ while its diffusivity in liquid water is obtained with Wilke-Chang’s correlation, eq 27,^39^ with viscosity (in cP) determined with the recommended correlation by the International Association for the Properties of Water and Steam.^40^
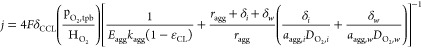
24
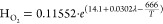
25
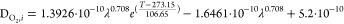
26
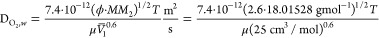
27

In summary, each model uses six fitting parameters: four dedicated to the activation description and two related to the ohmic losses. They are ξ_1_ (V), ξ_2_ (V/K), ξ_3_ (V/K), ξ_4_ (V/K), λ (−) and *ASR*_*other*_ (Ω m^2^) for the model with Amphlett’s description and *r*_*agg*_ (m), *f*_*Pt*_ (mass fraction), *φ*_*i*,*agg*_ (volumetric fraction), *α*_*cat*_ (−), λ (−) and *ASR*_*other*_ (Ω m^2^) for the other.

### Parameter Fitting and Comparison Strategy

To fit the parameters of the two developed models, an optimization problem is proposed. The objective function to be minimized, presented in eq 28, is the squared error between experimental voltage data and model predictions. Note that, as a consequence of the semiempirical nature of the model, experimental data is demanded to fit parameters with a physical meaning behind them. So, the quality of the used data directly affects its accuracy.
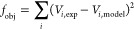
28

As for the constraints, all parameters have a lower and upper boundary. They are related to their physical meaning and are necessary to limit the solution to values representative of reality. The ones for the first model are assumed to be equal to the practical boundaries proposed by Blanco-Cocom e*t al.*^41^ and Shaheen et al.^23^ They are among the widest intervals found in the literature for those parameters, so it is expected that the best reasonable fits will be obtained. In those sources, resistance – and not area-specific resistance – is used, so their values were converted considering the area of 64 cm^2^ for the BCS 500 W fuel cell used by Shaheen. They are presented in Table 2.

**Table 2:** Boundaries for the Parameters of the First Model^a^

Parameter	Lower boundary	Upper boundary
ξ_1_ (V)	0.8532	1.1997
ξ_2_ (V/K)	–5.00 × 10^–03^	–1.00 × 10^–03^
ξ_3_ (V/K)	–9.80 × 10^–05^	–3.60 × 10^–05^
ξ_4_ (V/K)	9.54 × 10^–05^	2.60 × 10^–04^
λ (−)	10	23
*ASR*_*other*_ (ohm m^2^)	6.40 × 10^–07^	5.12 × 10^–06^

a Source: refs. (23,41)

Regarding the model that considers the agglomerate structure, the boundaries for *r*_*agg*_ and *φ*_*i*,*agg*_ are chosen as the highest and lowest values reported in the review of agglomerate parameters in the literature provided by Li et al.^35^ For *f*_*Pt*_, the value of the lower boundary is the lowest on the tests made by Khajeh-Hosseini-Dalasm et al.,^36^ while the highest is the value present in the works of Xu et al.^42^ and Fan et al.^37^ The transfer coefficient’s lower value is 0.5 based on the commonly observed low cathode potential values,^43^ while the higher one is taken from the work of Jiao et Li.^44^ It should be noted that Neyerlin et al.^43^ obtained accurate descriptions with *α*_*cat*_ = 1 when studying the oxygen reduction reaction, so values close to 1 are expected for this parameter. Finally, both other parameters follow the same reasoning as the previous model. Table 3 shows these intervals.

**Table 3:** Boundaries for the Parameters of the Second Model^a^

Parameter	Lower boundary	Upper boundary
*r*_*agg*_ (nm)	50	1500
*f*_*Pt*_ (−)	0.10	0.60
*φ*_*i*,*agg*_ (−)	0.10	0.60
*α*_*cat*_ (−)	0.50	2.00
λ (−)	10	23
*ASR*_*other*_ (ohm m^2^)	6.40 × 10^–07^	5.12 × 10^–06^

a Source: refs. (23, 35, 36, 42, 43, 44.)

Furthermore, three additional constraints must be used. The first, relative to *φ*_*i*,*agg*_, is obtained using eq 17 along with the fact that the thickness of the ionomer film cannot be smaller than zero. This yields eq 29. The other two are relative to *φ*_*Pt*/*C*_, stating that this value – evaluated with eq 13 – must be between 0 and 1.
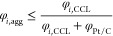
29

To solve this optimization problem, the genetic algorithm (GA) available in MATLAB’s global optimization toolbox is employed. In it, a population size of 500 is used, and the convergence criterion is when a relative change of less than 10^–12^ is observed in the last 200 iterations. The problem is solved using parallel computation with a Ryzen 7 2700 (∼3.2 GHz) processor and 16 GB of RAM.

The experimental data used in this work is the polarization results for a cell at seven different conditions presented by Dobson et al.^30^ Information about this cell is presented in Table 4. Note that the cell has a microporous layer, which is not considered in this work. Thus, it will be assumed to have the same properties as the gas diffusion layers. The cell’s pressure, inlet relative humidity, and temperature for each of the seven conditions are shown in Table 5.

**Table 4:** Physical Properties of the Analyzed Fuel Cell^a^

Parameter	Value
*δ*_*GDL*_ (μm)	250
*δ*_*MPL*_ (μm)	50
*δ*_*ACL*_ (μm)	3
*δ*_*CCL*_ (μm)	10
*δ*_*PEM*_ (μm)	25
*m*_*Pt*_ (mg/cm^2^)	0.40
*ε* (−)	0.60
τ (−)	3.00^45^
*A*_*cell*_ (cm^2^)	48.4

a Source: ref. (30)

**Table 5:** Cell’s Operating Conditions^a^

Data set	Pressure (kPa)	RH(%)	Temperature (K)
1	101.3	70%	353.15
2	101.3	50%	353.15
3	202.6	70%	353.15
4	202.6	50%	353.15
5	101.3	50%	368.15
6	202.6	70%	368.15
7	202.6	50%	368.15

a Source: ref. (30)

Moreover, the additional parameters for the second model are presented in Table 6. Between them, *φ*_*i*,*CCL*_ and *a*_*eff*_ are expected to vary in different cells, so they have to be assumed based on literature values if measurements are not available. Naturally, this is a limitation of the proposed model.

**Table 6:** Additional Parameters Used in the Second Model^a^

Parameter	Value	Source
*ε*_*i*,*CCL*_ (−)	0.30	(30)
*a*_*eff*_ (−)	70%	(46)
*ρ*_*Pt*_ (kg m^–3^)	21450	(35)
*ρ*_*C*_ (kg m^–3^)	1800	(35)
*E*_*act*,*cat*_ (J mol^–1^)	67000	(43)
(A cm^–2^Pt)	2.47 × 10^–08^	(43)
*s* (−)	0.10	(47)

a Source: refs. (30,35,43,46,47)

**Figure 1:**
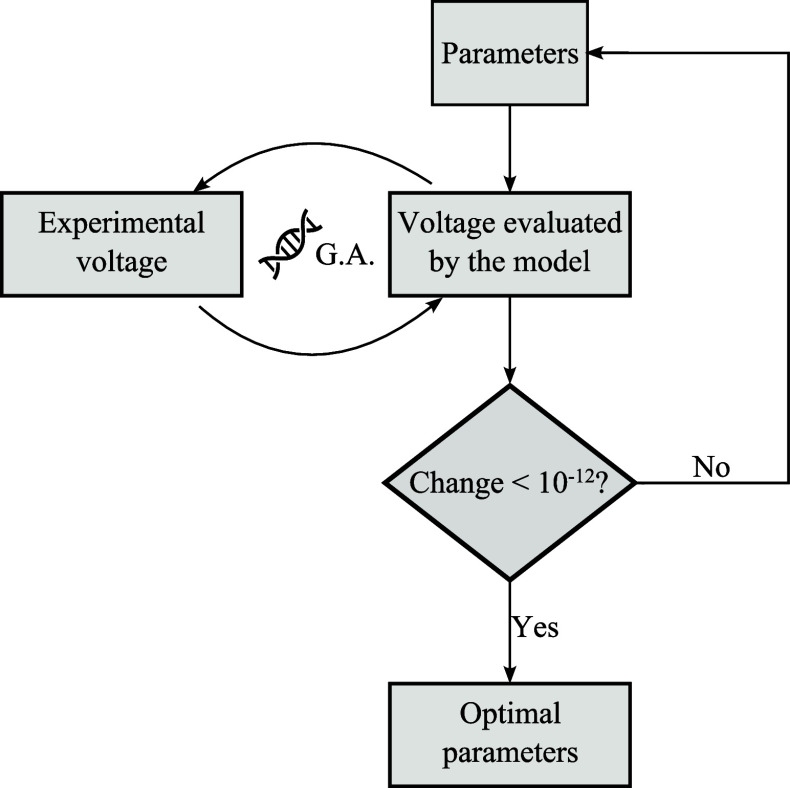
– Flowchart of the fitting procedure.

Finally, to evaluate both of those models, two distinct fitting procedures are used. This is done to compare the models in their capacity to describe experimental data and in the robustness of the estimated parameters. In the first, the models are fitted individually to each of the seven conditions, and in the second, they are fitted to all data at once. In this last, although the activation parameters are the same in all conditions, each will have its λ and *ASR*_*other*_ because the humidity and temperature may significantly alter them. In other words, the second case uses 18 parameters in the optimization: the four activation ones and two specifics for each condition to describe the ohmic losses. A flowchart presenting this parameter fitting procedure is displayed in Figure 1.

The quality of those fits is evaluated qualitatively with the plots and also quantitatively with the root-mean-square error between experimental data and model values. It should be noted that, as the root-mean-square error only differs from the objective function by a division by the number of used experimental points and a square root, the same optimal parameters would be obtained using it as the objective function. So, besides being more palatable information than the direct output of eq 28, it is an accurate representation of the accuracy of the fit.

## Results and Discussion

**Figure 2:**
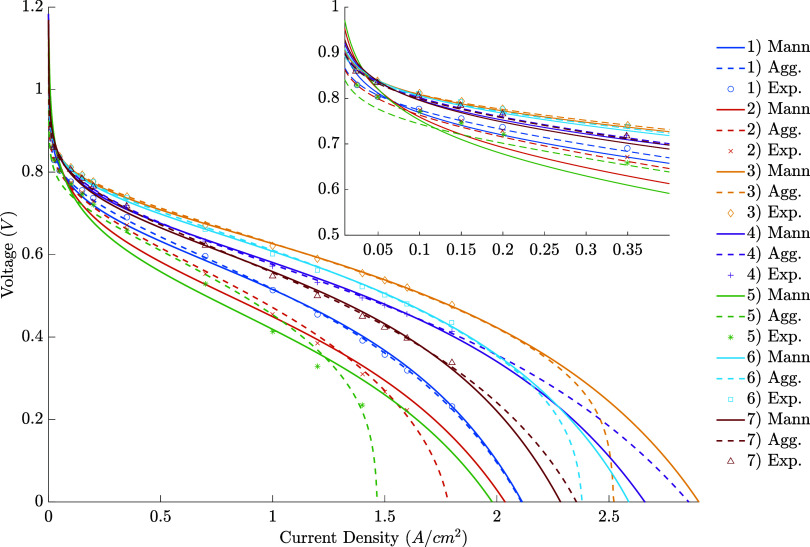
– Polarization curves for the individual fits.

Starting with the tests that individually fit each condition presented in Table 5, the polarization curves and root-mean-square error are presented in Figure 2 and Table 7. To ease the visualization, the same data is displayed in Figures S1 and S3 but separated between data sets at 353.15 and 368.15 K. This is also done for the other figures presented in this work. The optimization for obtaining the parameters at each condition of the first model took about 40 s, while times close to 30 min were necessary for the second. However, with the optimized parameters, both models could run 1000 current densities in less than 1 s, indicating that the more complex model also has good computational efficiency.

**Table 7:** RMSE for the Individual Fits^a^

Condition	RMSE (V) First model	RMSE (V) Second model	Improvement with the second model
1	1.239 × 10^–02^	4.624 × 10^–03^	62.68%
2	2.899 × 10^–02^	1.152 × 10^–02^	60.26%
3	3.983 × 10^–03^	3.304 × 10^–03^	17.03%
4	8.975 × 10^–03^	3.942 × 10^–03^	56.08%
5	3.913 × 10^–02^	2.638 × 10^–02^	32.59%
6	6.959 × 10^–03^	6.097 × 10^–03^	12.39%
7	1.284 × 10^–02^	6.562 × 10^–03^	48.88%

a Source: the author.

A qualitative analysis of the curves indicates that both models have good fits for most conditions. The most notable exceptions are that the first model (Amphlett’s approach) has a significantly worse description for condition 2, especially at higher current densities, and that neither model could provide a good fit for condition 5. In general, the second model fits the initial points better, reinforcing that describing the agglomerate structure leads to better prediction of the activation overvoltage. Moreover, although the fit in the final experimental points is approximately of equal quality between both models, the behavior after those points differs significantly. In the majority (2, 3, 5, and 6), the agglomerate model predicts a sharper voltage drop, which is reasonable because it considers resistances present at the agglomerate scale. Nevertheless, the other cases (1, 4, and 7) either do not present a significant difference at those conditions or present the opposite behavior, with Mann’s model predicting lower overvoltages. A hypothesis for this behavior is presented further. Quantitatively evaluating the error, relevant improvements – some of more than 60% – are obtained when the second model is used, confirming the previous observations.

**Figure 3:**
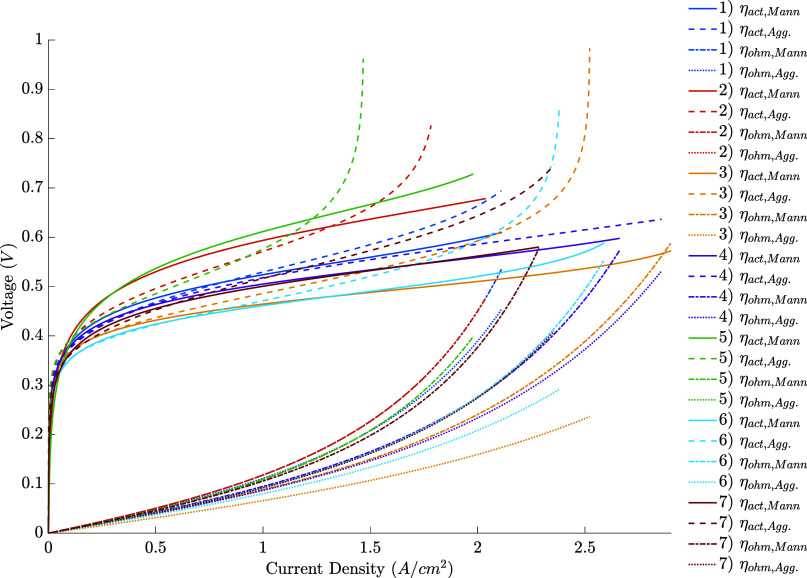
– Overvoltage results for the individual fits.

Another difference between both models can be seen in Figure 3 (Figures S2 and S4 for better visualization), displaying the overvoltage contribution predicted by them in each condition. The agglomerate models attribute a more significant part of the losses in higher current densities to the activation overvoltage. This explains why the second model predicts sharper voltage drops at higher current densities than the first: the agglomerate model is capable of describing the losses caused by oxygen depletion even without an additional concentration overvoltage term. The extra resistances present at the agglomerate level – associated, for example, with the solubilization and diffusion in the ionomer – cause an increase in the activation overvoltage. These resistances likely cause a considerable reduction of the oxygen concentration at the catalyst when compared to the simpler description, which explains the presence of a sharp increase in the activation overvoltage at higher current densities (lower voltages) in the proposed model, which are not seen using Mann’s approach. So, it is expected that the improvement caused by its usage will be even more significant if more points at higher current densities are fitted.

Note that even in cases such as condition 1, where the polarization curves have a similar behavior at higher current densities, the description of experimental data at the beginning of the domain is better with the agglomerate model and the attribution of the losses to each overvoltage is different. Therefore, the conclusion about the necessity of an agglomerate scale description for better representation points at lower voltages still stands, because even when a good fit is obtained without it, this likely comes at the cost of the quality of the parameters.

Furthermore, the optimal parameters for these tests are displayed in Table 8. Inspecting them, it is remarkable that the activation ones vary considerably between conditions. This is reasonable for ξ_2_, which has in its definitions the concentration of protons, water, and hydrogen at the catalyst layers, which are considered approximately constant by Amphlett et al.^13^ in its proposal but, in reality, can vary significantly between conditions. Nevertheless, the others – especially those of the second model – should be invariant because they represent more fundamental properties of the cell, which do not change with temperature, pressure, or humidity. This reinforces Dobson’s^30^ conclusion that fitting polarization data individually is not a good strategy for obtaining representative values for the cell. An explanation for this is that the same polarization curve can be obtained with different relations between ohmic and activation overvoltage, so there is no way to guarantee that the description that will better fit the data is representative of reality using only one curve.

**Table 8:** Parameters Obtained for the Individual Fit of the Experimental Data^a^

	Parameter	Condition 1	Condition 2	Condition 3	Condition 4	Condition 5	Condition 6	Condition 7
**First model**	ξ_1_ (V)	0.9783	0.8813	0.8587	0.9225	0.9105	1.0698	0.9477
	ξ_2_ (V/K)	–3.497 × 10^–03^	–3.289 × 10^–03^	–2.877 × 10^–03^	–3.239 × 10^–03^	–3.411 × 10^–03^	–3.625 × 10^–03^	–3.308 × 10^–03^
	ξ_3_ (V/K)	–9.799 × 10^–05^	–9.799 × 10^–05^	–8.687 × 10^–05^	–9.799 × 10^–05^	–9.800 × 10^–05^	–9.765 × 10^–05^	–9.800 × 10^–05^
	ξ_4_ (V/K)	1.532 × 10^–04^	2.264 × 10^–04^	1.078 × 10^–04^	1.417 × 10^–04^	2.462 × 10^–04^	1.118 × 10^–04^	1.459 × 10^–04^
	λ (−)	10.00	10.00	15.09	13.38	10.00	12.58	10.42
	*ASR* (Ω m^2^)	5.120 × 10^–06^	5.110 × 10^–06^	5.107 × 10^–06^	5.116 × 10^–06^	5.113 × 10^–06^	5.108 × 10^–06^	5.116 × 10^–06^
**Second model**	*r*_*agg*_ (nm)	1165	1495	335.1	1135	632.8	266.3	1490
	*f*_*Pt*_ (−)	0.2528	0.2638	0.3461	0.1845	0.3009	0.3858	0.2472
	*φ*_*i*,*agg*_ (−)	0.3070	0.3127	0.1000	0.1242	0.2725	0.1000	0.3009
	*α*_*cat*_ (−)	0.9378	0.9061	0.9236	0.8676	0.8836	0.9303	0.8680
	λ (−)	10.71	10.00	23.00	15.55	10.00	16.43	12.67
	*ASR* (Ω m^2^)	5.109 × 10^–06^	5.118 × 10^–06^	4.401 × 10^–06^	5.107 × 10^–06^	5.114 × 10^–06^	5.114 × 10^–06^	5.112 × 10^–06^

a Source: the author.

As for the ohmic-related parameters, in most cases, λ is close to the lower interval while *ASR*_*other*_ is almost equal to the higher one. Both of these values maximize the cell’s ohmic overvoltage. Two hypotheses are given to explain this, which are not mutually exclusive. The first is that Mann’s semiempirical relation for resistivity is insufficient to describe the ohmic losses in this fuel cell. This could be the case either because it was not developed considering Nafion NRE-211, used in the studied cell, or because the λ profile inside the membrane is not sufficiently well described by a single value. The other hypothesis is that voltage losses that are related to other effects are being described by ohmic losses. This is reinforced by the fact that the second model, which uses a more robust approach to describe activation losses, predicts higher λ values than the first. So, effects like the voltage losses caused by oxygen depletion – better described by agglomerate models – may be indirectly affecting the performance of those parameters. However, it should be highlighted that effects related to temperature variation and liquid water presence are also being neglected by the second model, so it is too attributing erroneously some part of it to the ohmic losses. Even with these reservations, the ohmic parameters are consistent between cases. For example, all conditions that only vary in relative humidity present a higher λ in the more humidified condition. Therefore, even if the individualized fits may hinder the parameters, they still follow their physical meaning.

**Figure 4:**
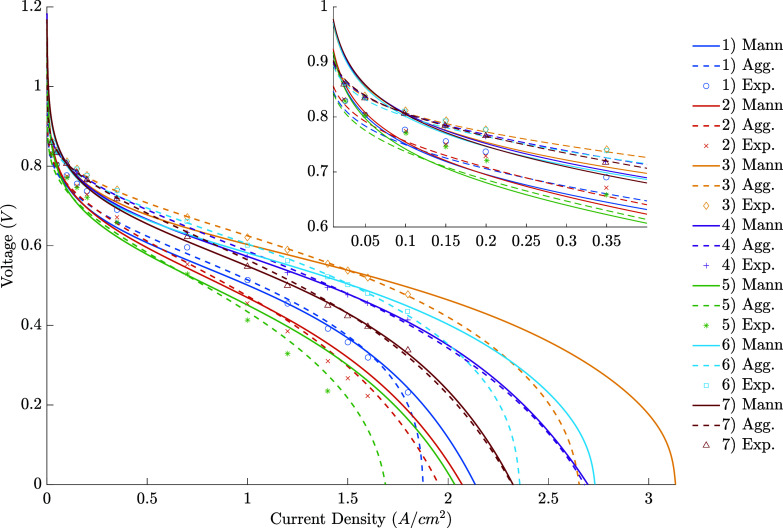
– Polarization curve of the simultaneous fit.

The fits made considering all the conditions simultaneously are shown in Figure 4 (Figures S5 and S7 ), with the RMSE for them presented in Table 9. It took about 60 s to fit the first model and 2.5 h for the second. As expected, the errors here are more significant than the individual fits, but the description is still reasonable. Again, the second model has a better fit of the data, indicating that it not only has a better capacity for fitting individual polarization curves but is also more robust in its description. Examples in which this difference is evident are conditions 3 and 6, where the first model underestimates the data in smaller current densities and overestimates them in the last points, while the second has a good agreement throughout the curve.

**Table 9:** RMSE for the Simultaneous Fits^a^

Condition	RMSE (V) First model	RMSE (V) Second model	Improvement with the second model
1	2.459 × 10^–02^	2.186 × 10^–02^	11.10%
2	3.385 × 10^–02^	2.069 × 10^–02^	38.88%
3	2.139 × 10^–02^	3.378 × 10^–03^	84.21%
4	1.712 × 10^–02^	8.492 × 10^–03^	50.40%
5	4.686 × 10^–02^	3.009 × 10^–02^	35.79%
6	2.355 × 10^–02^	7.569 × 10^–03^	67.86%
7	2.000 × 10^–02^	9.839 × 10^–03^	50.81%

a Source: the author.

In contrast to what was observed in the individual fit, here the second model always predicts larger losses at high current densities, which is coherent with the additional activation resistances that are described. This reinforces the previous argument that fitting an individual polarization curve is insufficient to obtain parameters that are representative of the cell. So, the behavior observed in the individual fit is likely related to fits that, while mathematically better, do not represent well the real parameters.

A noteworthy result is that the mean RMSE for the simultaneous fit of the second model (1.46 × 10^–02^) is smaller than the mean RMSE for the individual fit of the first model (1.62 × 10^–02^). This is also true for a case-by-case comparison for 5 of the 7 test conditions. So, the second model is on average still better than the first at more restrictive conditions, in which the parameters are expected to be more meaningful. This observation reinforces the hypothesis that using an agglomerate model to describe the activation can improve semiempirical models.

Furthermore, the fitted parameters for this test are presented in Table 10. As for the other fit, both models’ λ follow what is expected for the system, that is, under the same pressure and temperature, higher values occur when the humidity is higher. Thus, the ohmic overvoltage description, even if the caveats stated for the individual fit are still true, is consistent with the expected behavior.

**Table 10:** – Parameters Obtained for the Simultaneous Fit of All Experimental Dataa

First model (Amphlett)		Second model (Agglomerate)	
Parameter	Value	Parameter	Value
ξ_1_ (V)	0.8532	*r*_*agg*_ (nm)	1489
ξ_2_ (V/K)	–3.147 × 10^–03^	*f*_*Pt*_ (−)	0.2930
ξ_3_ (V/K)	–9.800 × 10^–05^	*φ*_*i*,*agg*_ (−)	0.3436
ξ_4_ (V/K)	1.956 × 10^–04^	*α*_*cat*_ (−)	0.8925
*λ*_*1*_ (−)	10.00	*λ*_*1*_ (−)	12.55
*ASR*_1_ (Ω m^2^)	8.948 × 10^–07^	*ASR*_1_ (Ω m^2^)	6.400 × 10^–07^
λ_2_ (−)	10.00	λ_2_ (−)	10.00
*ASR*_2_ (Ω m^2^)	5.120 × 10^–06^	*ASR*_2_ (Ω m^2^)	5.106 × 10^–06^
λ_3_ (−)	20.77	λ_3_ (−)	16.34
*ASR*_3_ (Ω m^2^)	6.400 × 10^–07^	*ASR*_3_ (Ω m^2^)	6.470 × 10^–07^
λ_4_ (−)	13.46	λ_4_ (−)	15.99
AS*R*_4_ (Ω m^2^)	2.218 × 10^–06^	AS*R*_4_ (Ω m^2^)	5.116 × 10^–06^
λ_5_ (−)	10.00	λ_5_ (−)	10.00
*ASR*_5_ (Ω m^2^)	5.120 × 10^–06^	*ASR*_5_ (Ω m^2^)	5.107 × 10^–06^
λ_6_ (−)	15.59	λ_6_ (−)	14.41
*ASR*_6_ (Ω m^2^)	6.400 × 10^–07^	*ASR*_6_ (Ω m^2^)	6.490 × 10^–07^
λ_7_ (−)	10.64	λ_7_ (−)	11.39
*ASR*_7_ (Ω m^2^)	2.702 × 10^–06^	*ASR*_7_ (Ω m^2^)	5.120 × 10^–06^

a Source: the author.

Regarding the activation parameters, ξ_1_ and ξ_3_ have values equal to the lower boundaries. This may indicate that the commonly used practical boundaries for them are not representative of the studied cell, even though they are among the widest reported in the literature. However, it is difficult to conclude due to the number of physical parameters lumped together in each ξ. This is not a problem in the second model, where it is clear that each parameter is reasonable when compared to expected values in fuel cells. The most noteworthy is the agglomerate radius because it is closer to the upper limit of the interval. This may not be a problem because agglomerates with between 1 and 5 μm were observed by Broka and Ekdunge^48^ with scanning electron microscopy (SEM). Nevertheless, considering that the increase in the radius increases the activation overvoltage, this may be caused by the necessity of compensating for the imperfect ohmic overvoltage description or other resistances caused by nonmodeled factors, such as liquid water presence. Therefore, the second model is promising not only to obtain a more accurate fit but also to have more meaningful parameters. However, it is possible that a more accurate description of the ohmic effects is necessary to obtain values closer to the real ones.

**Figure 5:**
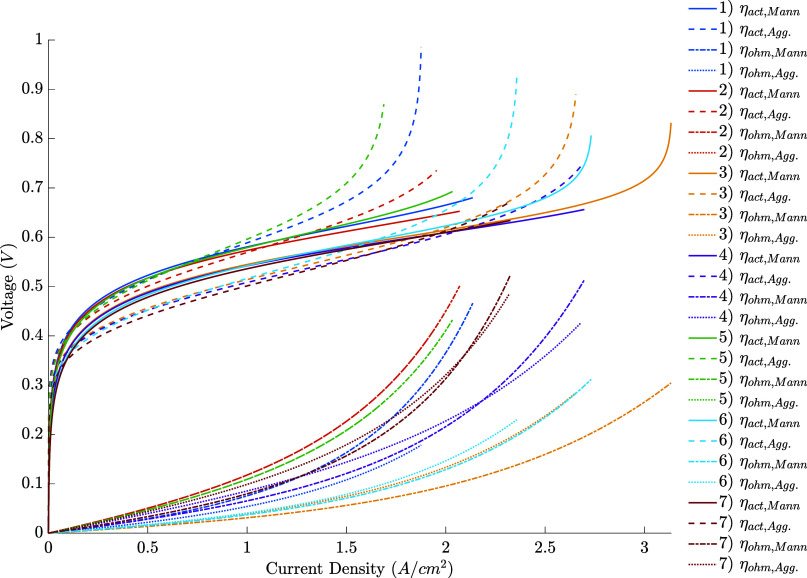
– Overvoltage results of the simultaneous fit.

Lastly, the contribution of the overvoltage in each condition with the simultaneous fit is displayed in Figure 5 (Figures S6 and S8). A considerable difference exists between how each model attributes the overvoltage. The activation overvoltage is higher in the second model, with significant differences appearing at higher current densities. As a consequence, the first model predicts a higher contribution of ohmic effects to the total losses. Without experimental measurements of each overvoltage’s contribution – which could be made using Electrochemical Impedance Spectroscopy – a conclusion about the accuracy of those predictions cannot be reached. Nevertheless, based on the better fit of the polarization curves in this work, previous works stating that agglomerate models have good descriptions of the activation overvoltage and the usage of a thin membrane and catalyst layers in the analyzed cell, the second model is more likely to be accurately representing the real fuel cell behavior.

## Conclusion

Based on the qualitative and quantitative results obtained, the use of an agglomerate model to describe activation losses in semiempirical PEMFC models proves to be a promising alternative, offering superior fits to experimental polarization data compared to the commonly employed Amphlett approach. Additionally, the proposed model has as advantage the usage of fitting parameters that, while not always readily available for fuel cells, offer a clear physical interpretation. This enhances the ease of analyzing optimization results and defining appropriate parameter boundaries.

Another noteworthy feature of the agglomerate model implementation is its tendency to attribute a larger portion of the overvoltage to the activation process, especially at higher current densities. While further analysis is required for more definitive conclusions, these findings suggest that the model is capable of capturing oxygen depletion effects. This highlights the importance of effects at the agglomerate scale to the overall resistance present in the cell. As a consequence, not only the obtained fit is better, but also the attribution of the losses to each overvoltage is likely more exact. Thus, it may be able to effectively describe the full range of polarization results across multiple conditions using the same activation parameters, attesting to its robustness.

The primary limitation of this approach is the significantly higher computational demand required for parameter fitting compared to simpler catalyst layer descriptions. Nevertheless, once the optimal parameters are determined, both models exhibit excellent computational efficiency, solving 1000 data points in under one second on a standard personal computer.

Therefore, if the computational time required for parameter fitting is not a limiting factor, agglomerate models for catalyst layers in semiempirical models are recommended for achieving more accurate and meaningful fits. However, given that the simpler Amphlett model produced satisfactory results under most conditions with considerably lower computational demands, it remains a viable alternative, provided that transport-related losses are not critical for the studied conditions.

## Supporting Information Available

Polarization and global overvoltage curves separated by condition to ensure easier visualization (PDF)

## Nomenclature

*V*: voltage, V
*r*_*agg*_: agglomerate radius, m
*E*_*thermo*_: thermodynamic voltage, V
δ: layer thickness, m
*T*: temperature, K
*N*_*agg*_: agglomerate density, agglomerates m^–3^
*P*: pressure, Pa
*k*_agg_: reaction constant, s^–1^
*p*: partial pressure, Pa
α: transfer coefficient
*j*: current density, A m^–2^
*ϕ*_*agg*_: Thiele modulus
*j*_*lim*_: limiting current density, A m^–2^
*E*_*agg*_: effectiveness factor
*j*_0_: exchange current density, A cm^–2^Pt
*H*: Henry constant, Pa m^3^ mol^–1^
η: overvoltage, V
*R*: ideal gas constant,
J mol^–1^ K^–1^: *Subscript*
*F*: Faraday constant, s A mol^–1^
act: activation
*D*: diffusion coefficient, m^2^ s^–1^
ohm: ohmic
*MM*: molar mass, kg kmol^–1^
conc: concentration
*s*: liquid water saturation
ref: reference
ϵ: porosity
Pt/C: solid phase of the agglomerate
τ: tortuosity
i: ionomer
*ASR*: area-specific resistance, Ω m^2^
λ: semiempirical parameter representing membrane humidification*Superscripts*
ξ: semiempirical parameter related to activation, V or V K^–1^
eff: effective value
ρ: density, kg mol^–1^, or resistivity,
Ω m: *Acronyms*
φ: volumetric fraction
*PEMFC*: proton-exchange membrane fuel cell
*C*: concentration, mol cm^–3^ or mol m^–3^
*GDL*: gas diffusion layer
*m*_*Pt*_: catalyst loading, mg Pt cm^–2^ or kg Pt m^–2^
*MPL*: microporous layer
*f*_*Pt*_: platinum ratio in the Pt/C
CL: catalyst layer
catalyst-specific area, cm^2^ Pt m^–3^ of electrode
*PEM*: proton-exchange membrane

## Abbreviations

PEMFC: Proton-exchange membrane fuel cell
GDL: gas diffusion layer
MPL: microporous layer
CL: catalyst layer
PEM: proton-exchange membrane.
