# Supplementary material for: Comparison between Different Activation Overvoltage Descriptions for Semiempirical Proton-Exchange Membrane Fuel Cell Models
Source: ACS Omega. 2025 Apr 10;10(15):15381–92. doi: 10.1021/acsomega.4c11603 (PMC12019751; doi:10.1021/acsomega.4c11603)
Supplement: Supplementary file 1 — ao4c11603_si_001.pdf [file ao4c11603_si_001.pdf]

SUPPLEMENTARY MATERIAL FOR: COMPARISON BETWEEN DIFFERENT ACTIVATION OVERVOLTAGE DESCRIPTIONS FOR SEMI-EMPIRICAL PROTON-EXCHANGE MEMBRANE FUEL CELL MODELS

Leonardo F. Carneiro[a,*], Nicolas T. D. Fernandes[c], Esly F. Costa Junior[a,b], Tulio Matencio[a,c]

[a]*Postgraduate Program in Chemical Engineering, PPGEQ-UFMG, Federal University of Minas Gerais, Presidente Antônio Carlos Avenue, 6627, Pampulha, 31270-901, Belo Horizonte, Minas Gerais, Brazil*

[b]*Postgraduate Program in Mechanical Engineering, PPGMEC-UFMG, Federal University of Minas Gerais, Presidente Antônio Carlos Avenue, 6627, Pampulha, 31270-901, Belo Horizonte, Minas Gerais, Brazil*

[c]*Department of Chemistry, DEQ/ICEX-UFMG, Federal University of Minas Gerais, Presidente Antônio Carlos Avenue, 6627, Pampulha, 31270-901, Belo Horizonte, Minas Gerais, Brazil*

[*]*E-mail: fortunaleo@ufmg.br*

Individual fits:

T = 353.15 K:

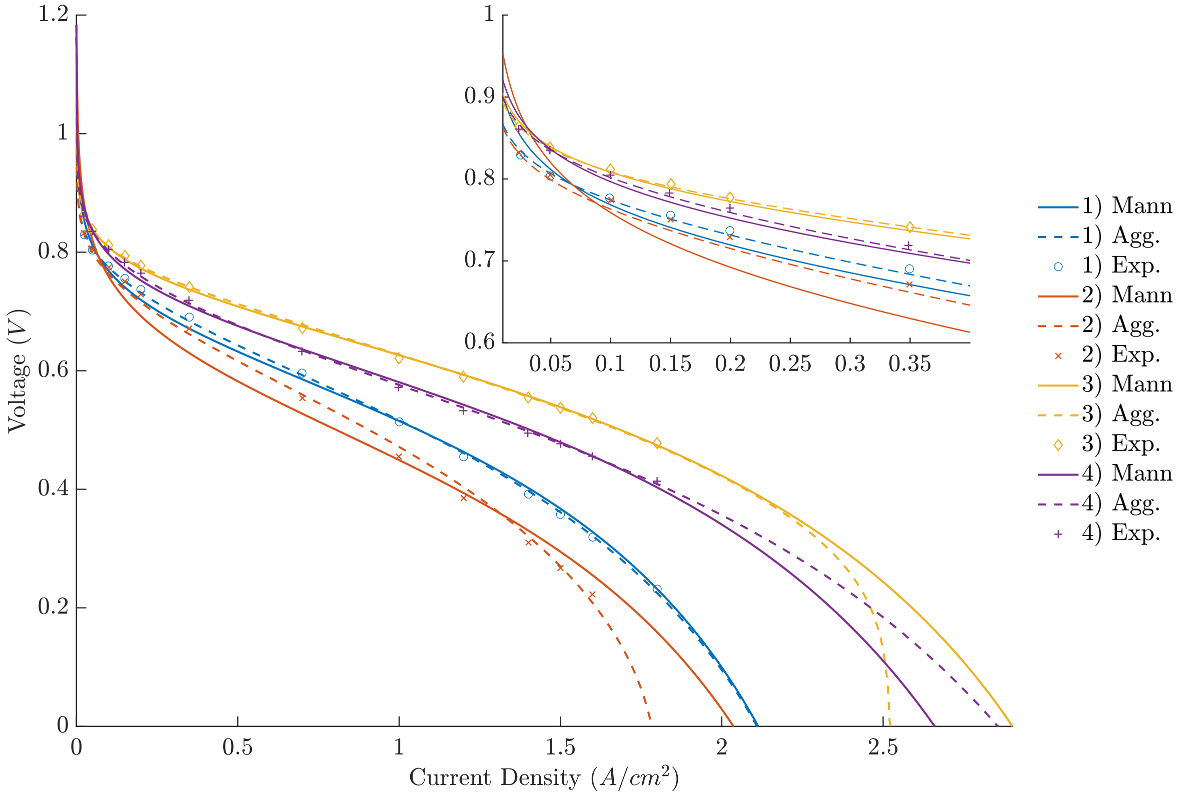

Figure S1 - Polarization curves of the individual fits for datasets at 353.15 K.

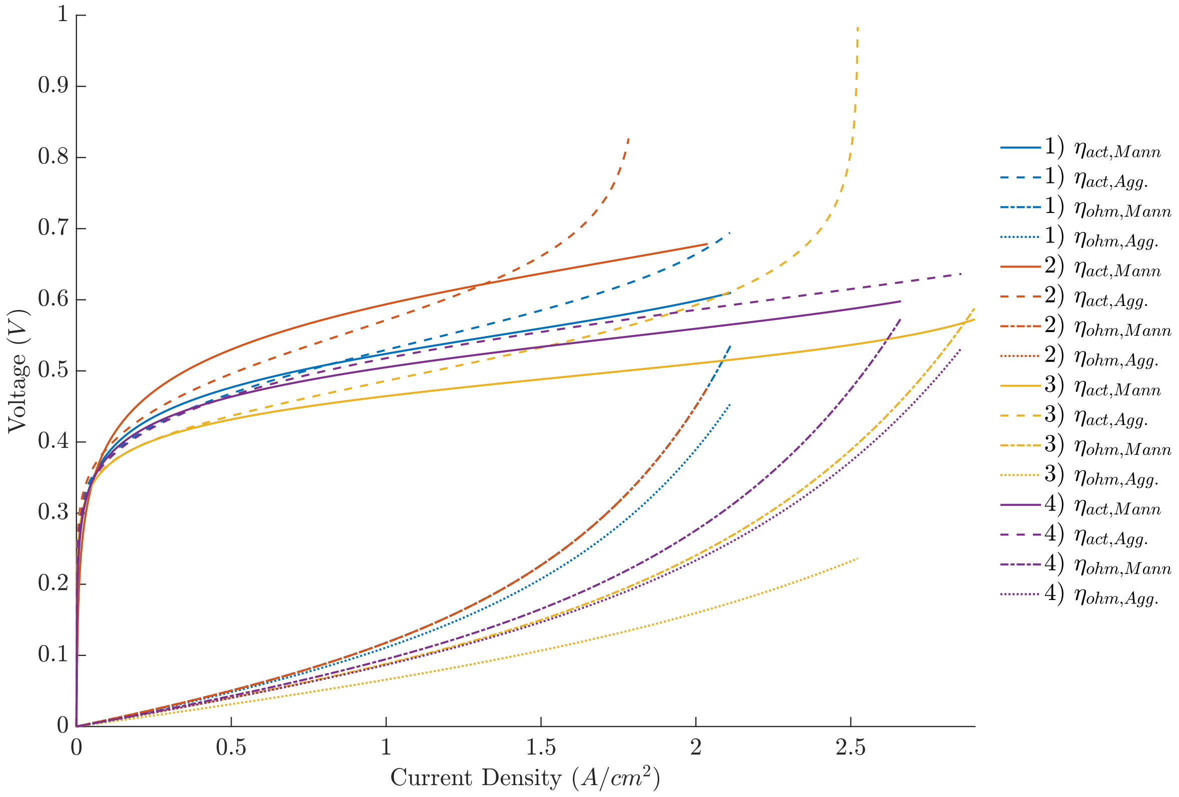

Figure S2 – Overvoltages of the individual fits for datasets at 353.15 K.

T = 368.15 K:

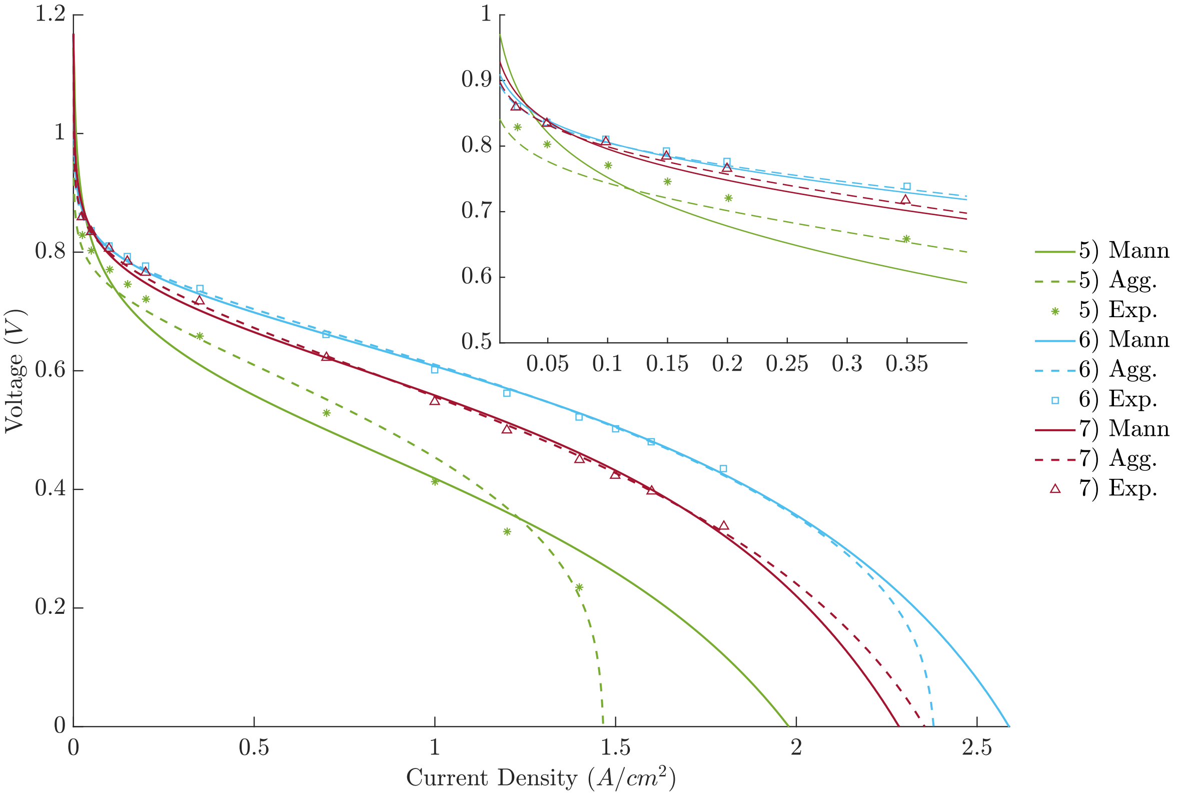

Figure S3 – Polarization curves of the individual fits for datasets at 368.15 K.

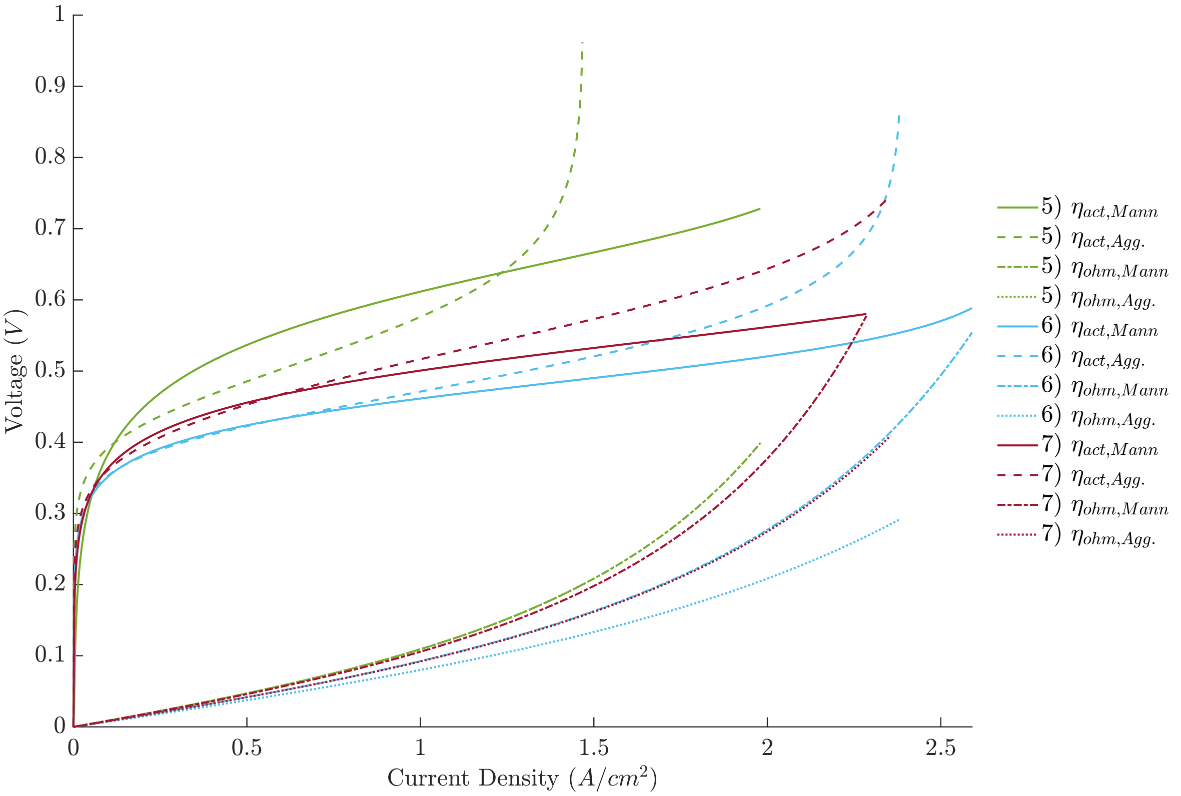

Figure S4 – Overvoltages of the individual fits for datasets at 368.15 K.

Simultaneous fits:

T = 353.15 K:

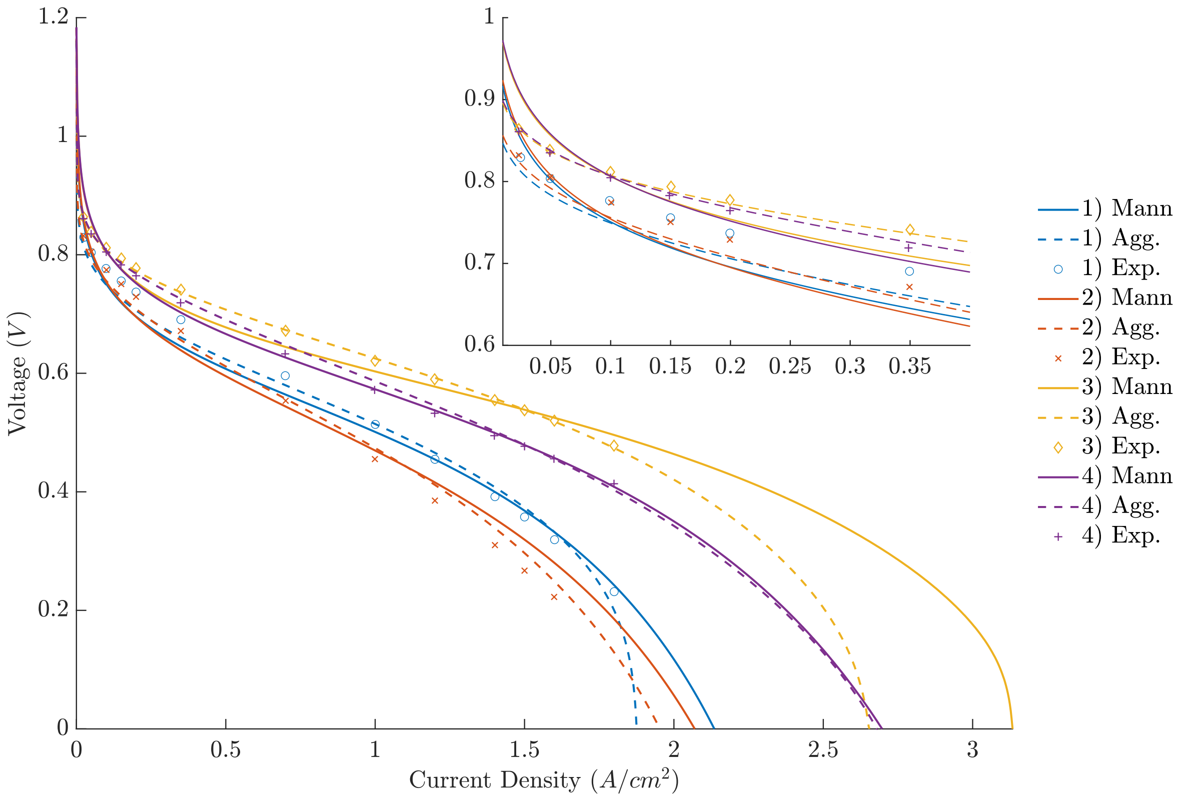

Figure S5 - Polarization curves of the simultaneous fits for datasets at 353.15 K.

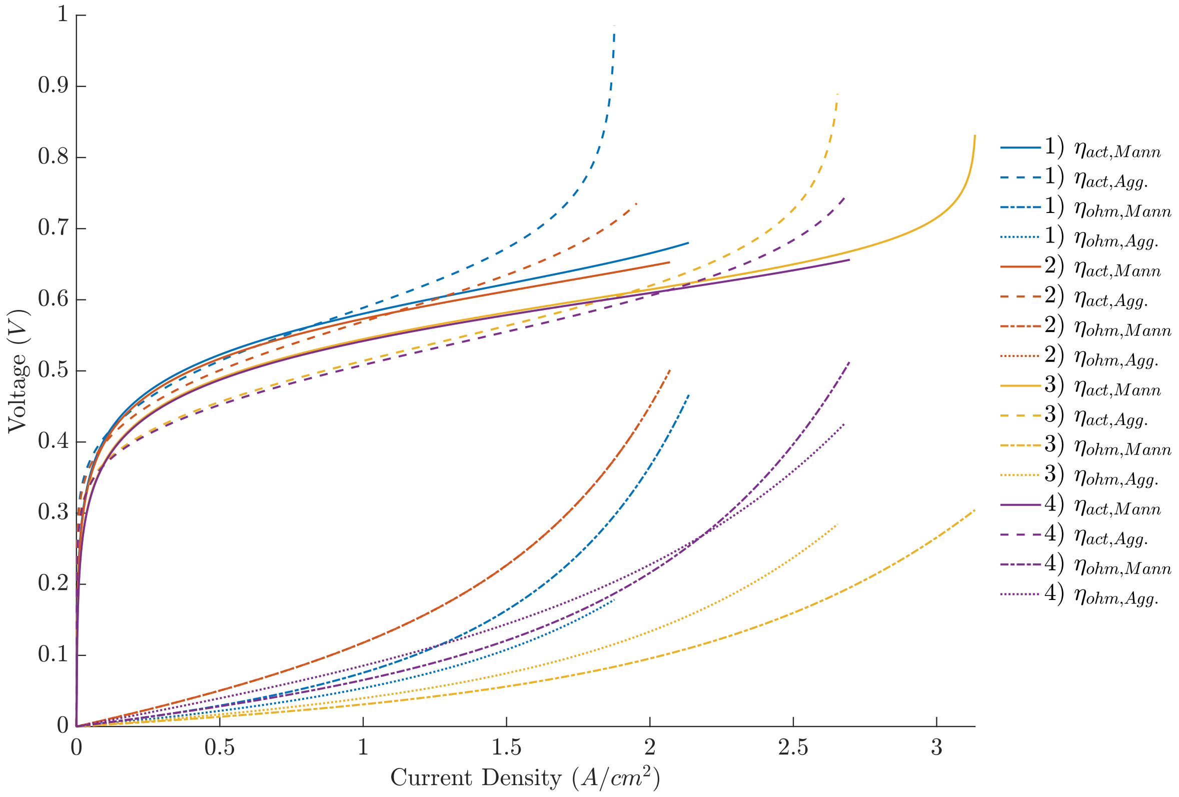

Figure S6 – Overvoltages of the simultaneous fits for datasets at 353.15 K.

T = 368.15 K:

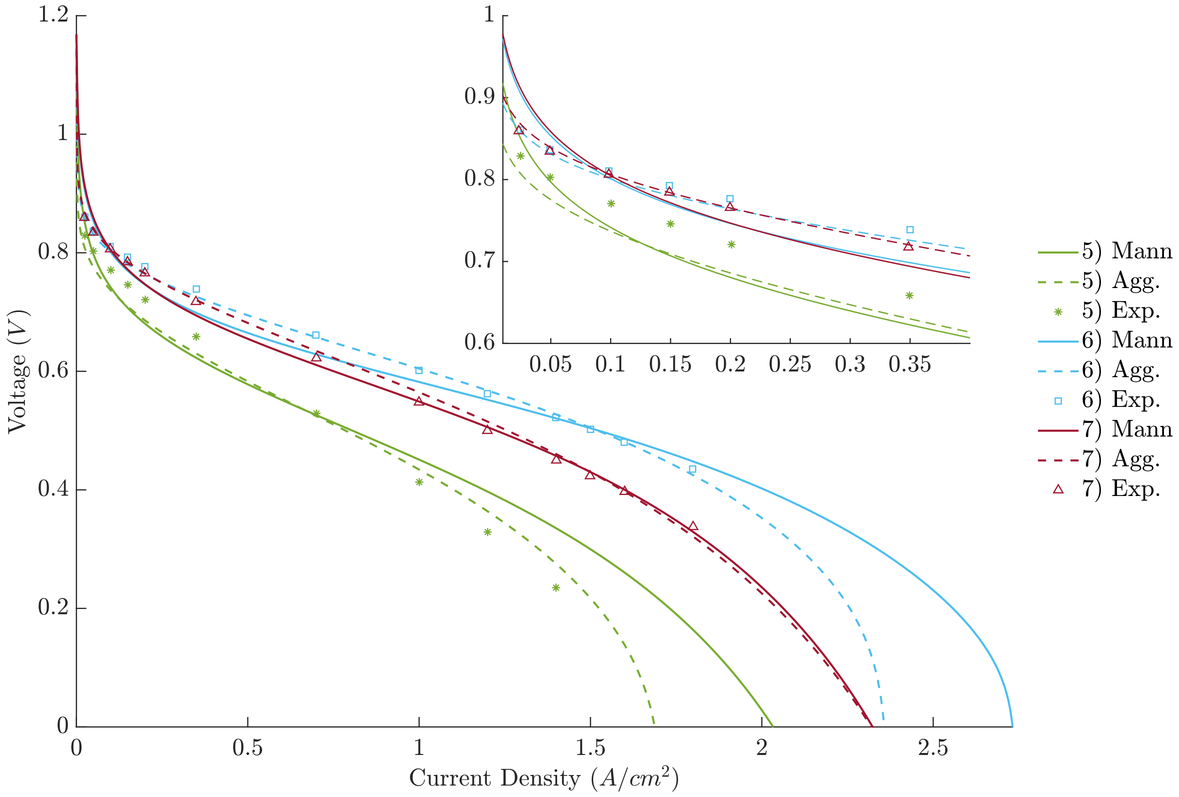

Figure S7 - Polarization curves of the simultaneous fits for datasets at 368.15 K.

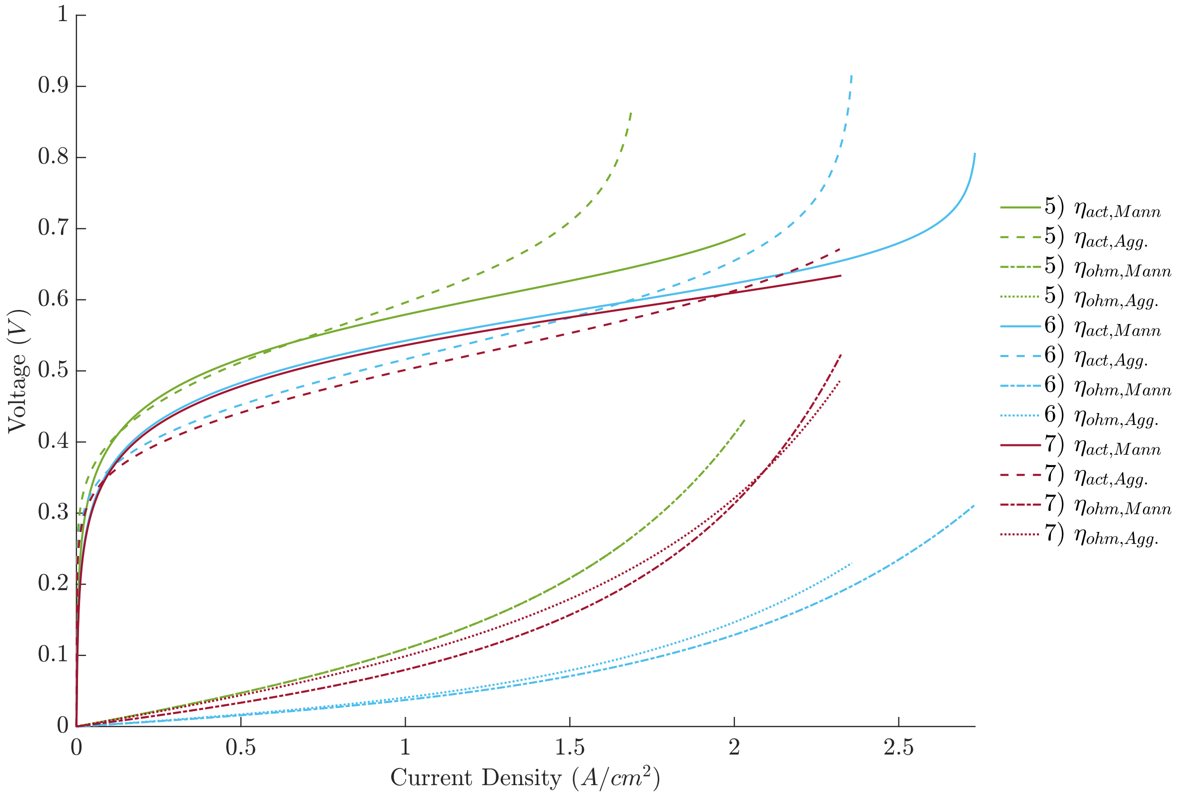

Figure S8 – Overvoltages of the simultaneous fits for datasets at 368.15 K.
